# ﻿Two new species of the genus *Acherontiella* (Collembola, Hypogastruridae) from caves of the Alborz and Kopet Dagh mountains in Iran

**DOI:** 10.3897/zookeys.1255.164170

**Published:** 2025-10-09

**Authors:** Mahmood Mehrafrooz Mayvan, Ľubomír Kováč

**Affiliations:** 1 Department of Zoology, Institute of Biology and Ecology, Faculty of Science, Pavol Jozef Šafárik University, Šrobárova 2, 041 54 Košice, Slovakia Pavol Jozef Šafárik University Košice Slovakia

**Keywords:** Morphology, setal pattern, Southwest Asia, subterranean fauna, troglobiont

## Abstract

Two new subterranean species of the genus *Acherontiella* Absolon, 1913 from Iran are described, illustrated, and morphologically differentiated from similar species. *Acherontiella nezamdoosti***sp. nov.** has larger distribution range covering caves of North Khorasan, Razavi Khorasan, and Tehran provinces, while *A. palaciosi***sp. nov.** is probably restricted to Moghan Cave in Razavi Khorasan province. Both new species are characterized by absence of eyes, PAO, empodium, furca, and retinaculum. *Acherontiella nezamdoosti***sp. nov.** is distinguished from other species by having two small anal spines, Th II–III with setae m1, m4, and m5, and four thickened anterior setae on the labrum. *Acherontiella palaciosi***sp. nov.** is characterized by bearing two medial setae in the anterior row of the labrum thickened into spines and apically dentate, and by the absence of setae m3 on Th I and p3 on Th II–III. An updated key and a table summarizing diagnostic characters of world species of the genus *Acherontiella* are provided.

## ﻿Introduction

*Acherontiella* Absolon, 1913 is a cosmopolitan genus belonging to the family Hypogastruridae and described from a cave in Algeria (Absolon 1913; Thibaud 1990). Species of this genus are characterized by their fusiform, unpigmented body, and the absence of eyes, post-antennal organ, retinaculum, and furca (Thibaud 1990; Thibaud et al. 2004). A substantial number of species have been described from caves. To date, *A. bougisi* Cassagnau & Delamare Deboutteville, 1955 is the only species of the genus *Acherontiella* recorded from Iran, where it was found in the soil of wheat fields in Khuzestan province (Mehrafrooz Mayvan et al. 2023).

Iran is a semi-arid country in Southwest Asia covering approximately 1.64 million km^2^ and zoogeographically assigned to the Palearctic region (Lomolino et al. 2017; Azizi Jalilian et al. 2020; Mehrafrooz Mayvan et al. 2021). The Alborz Mountain range in the northern Iran has biodiversity-rich Hyrcanian forests, and the Kopet Dagh mountain range in northeastern Iran is especially rich in karst caves, the most common type of caves worldwide (Culver and Pipan 2009; Memariani 2020; Davoudnia et al. 2024). This contribution is based on recent speleobiological fieldwork conducted in caves of northern and northeastern Iran with very limited available data to subterranean fauna. In this paper, we report on the discovery of two new species of the genus *Acherontiella*. They were found in Iranian caves within the Alborz (Hyrcanian forests) and Kopet Dagh mountain ranges. Additionally, we provide an updated identification key and a table summarizing the diagnostic characteristics of world species of the genus *Acherontiella*.

## ﻿Material and methods

### ﻿Cave descriptions

Table 1

**Table 1.** List of Iranian caves with records of the new species of *Acherontiella.* Altitude is above mean sea level.

Cave name	Province	GPS Coordinates (Latitude, Longitude)	Length	Altitude (m a.s.l.)	Temperature (°C)	Relative humidity (%)
Aghol	Razavi Khorasan	36°33'36"N, 59°04'58"E	25	1445	—	—
Al	Razavi Khorasan	36°40'0.2"N, 59°39'19"E	75	1447	—	—
Bazangan	Razavi Khorasan	36°18'29"N, 60°22'19"E	204	1401	—	—
Bidak	North Khorasan	37°26'30"N, 57°13'14"E	80	1237	—	—
Burnik	Tehran	35°41'14"N, 52°41'24"E	4022	2000	8	87
Danial	Mazandaran	36°39'35"N, 51°10'53"E	2158	164	16	84
Kardeh	Razavi Khorasan	36°40'15"N, 59°40'37"E	425	1458	—	—
Kenan 1	Razavi Khorasan	36°33'58"N, 59°04'19"E	541	1451	—	—
Moghan	Razavi Khorasan	36°06'59"N, 59°22'06"E	500	2193	9	84

**Aghol Cave** is a very small cave on a mountain slope near Kenan Caves, in Razavi Khorasan province, close to Chenaran city (36°33'36"N, 59°04'58"E). It is at an altitude of 1,445 m a.s.l. The cave features a corridor that is 25 m long, with a high ceiling, and one corner contains a large amount of bat guano.

**Al Cave**, located in Razavi Khorasan province, is about 50 km east of Mashhad, near Kardeh village, in the southern section of the Kopet Dagh Mountains (36°40'0.2"N, 59°39'19"E). It is within the Mozdouran geological formation of northeastern Iran. The cave is at an elevation of 1,477 m a.s.l. and has two adjacent entrances on a mountain slope in the middle of a valley. The first, well-shaped entrance leads to two relatively large halls.

**Bazangan Cave** is in northeastern Iran, in the Sarakhs region of Razavi Khorasan province, 140 km northeast of Mashhad (36°18'29"N, 60°22'19"E). It is in the Kopet Dagh mountains at an altitude of 1,401 m a.s.l.. Its entrance is relatively large. From the opening, the cave continues downward in a gentle slope towards a lake at the bottom. The ceiling is rocky, while the floor is heavily covered with bat guano and bird (mostly pigeon) droppings. In some parts of the cave, the ceiling height reaches of 25 m. The cave extends for 204 m.

**Bidak Cave** is in North Khorasan province, 4 km southeast of Bidak village and 12 km from Bojnourd city in an open area (37°26'30"N, 57°13'14"E). The cave entrance is located at an elevation of 1,327 m a.s.l. It appears at ground level as a vertical shaft resembling a well, with a stone-walled opening approximately 6 m high. The cave extends for about 80 m in length, with the first 20 m descending steeply at an almost vertical angle, followed by a passage that leads to a corridor and a chamber at the terminal part of the cave.

**Figure 1.**
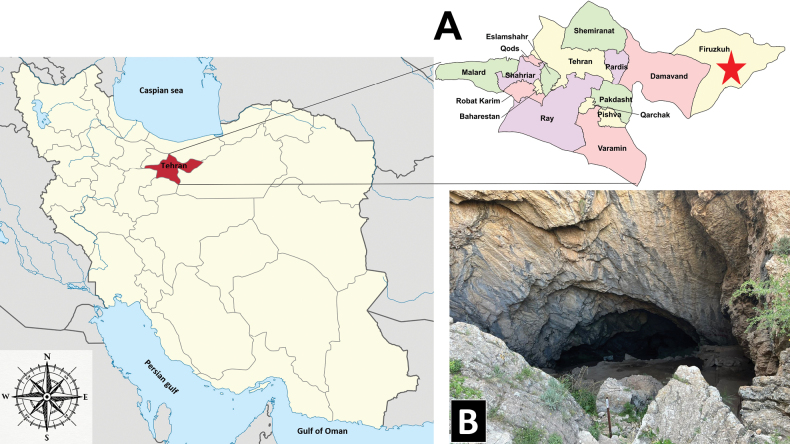
A. Location of Tehran province in Iran (red) and the Burnik Cave (red asterisk), type locality of *A. nezamdoosti* sp. nov.; B. Entrance of the Burnik Cave (photo: M. Mehrafrooz Mayvan).

**Figure 2.**
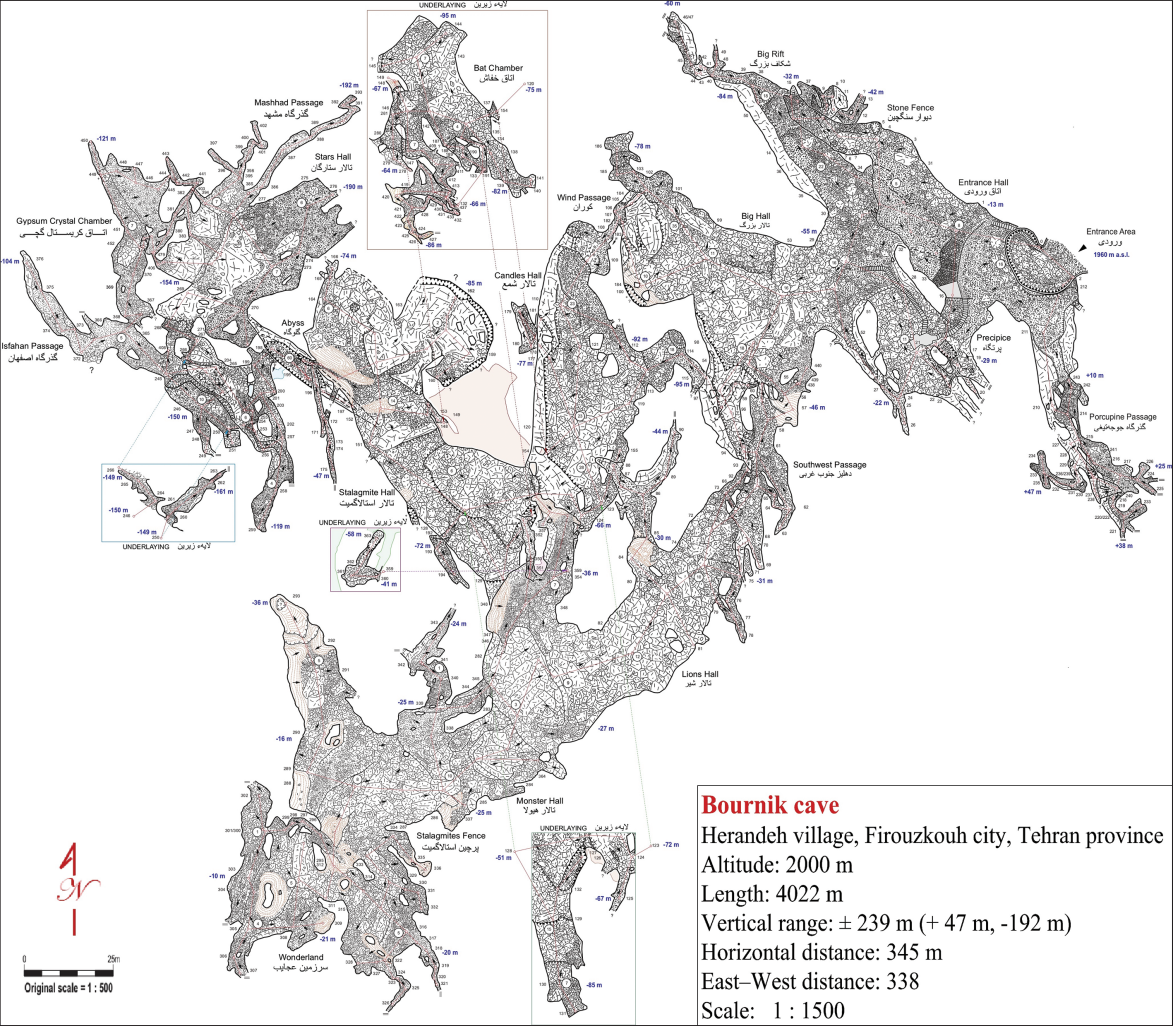
Ground plan of the Burnik Cave (sketched by E. Geyer).

**Burnik Cave** is within a limestone formation near the city of Firouzkouh and the village of Herandeh (35°41'14"N, 52°41'24"E) at an altitude of approximately 2,000 m a.s.l., about 100 m above the Burnik plain (Fig. 1). Its entrance is on a steep mountain slope and, at 4022 m, is the longest cave (Fig. 2) in Tehran province and the fourth longest cave in Iran. It features a large, triangular entrance, approximately 15 m long and 10 m high (Fig. 10A). The cave contains four extensive halls, with the Stalagmite Hall (Fig. 10B) containing a 30 m deep well near its towering cliffs. The temperature and relative humidity were measured in the terminal part of the cave in June 2024 using a TFA 30.5015 DTH thermo-hygrometer; the temperature was found to be 8 °C and humidity was 87%.

**Danial Cave** is in the Alborz Mountain range and the Hyrcanian Forest within the Mazandaran karst area on the southern edge of the Caspian Sea in Salman Shahr, Abbasabad city (36°39'35"N, 51°10'53"E). The cave is at an altitude of 164 m a.s.l. The cave has a length of 2,158 m, and a subterranean river flows throughout the cave’s entire length (Ghelich Khani 2017; Yusefinia 2017; Barjadze et al. 2024). It had constant air temperature of 16 °C and relative humidity over 84% in July 2024 (measured with a thermo-hygrometer TFA 30.5015 DTH).

**Kardeh Cave**, also known as Hezar Dalan (Thousand corridor), is 3 km from Kardeh village (36°40'15"N, 59°40'37"E) and 48 km from Mashhad city in Razavi Khorasan province. The entrance, located at an elevation of 1,458 m a.s.l., features a stone gap. The cave is 425 m in length.

**Kenan 1 Cave**, at an altitude of 1451 m a.s.l., is in the Zoukenan region, approximately 5 km south of Chenaran city in Razavi Khorasan province (36°33'58"N, 59°04'19"E). Although most of the cave remains dry, humidity can be observed in some areas during the rainy season. The cave is 541 m in length and is divided into two separate floors. It has six entrances and contains three wells.

**Moghan Cave** is 35 km southwest of Mashhad in Razavi Khorasan Province at an altitude of 2,193 m a.s.l. in eastern part of the Kopet Dagh Mountains (36°06'59"N, 59°22'06"E) (Figs 9, 10C). The cave is approximately 500 m long and has a maximum depth of 44 m. It features five chambers in two floors. There is a water pool in the terminal part of the fifth chamber on the second floor. This pool measures 5.66 × 6.93 m and has a depth of 0.3–0.6 m (Mehrafrooz Mayvan et al. 2024). The air temperature in this chamber is a constantly 9 °C, and the relative humidity exceeds 84% in July 2024 (measured by HTC-2 digital thermo-hygrometer).

### ﻿Methods

Collembola, including *Acherontiella* specimens, were collected cave walls, sediment, rotten wood, and bat guano using an aspirator. Pitfall traps equipped with plastic cups containing propylene glycol were used as a complementary collection method. Furthermore, from wooden shavings that were used as a bait exposed in a cave for about six months, the specimens were extracted in Berlese funnels in the laboratory of the Zoology Museum of Ferdowsi University of Mashhad in Iran. In the laboratory, the specimens were placed in containers filled with 96% ethyl alcohol for preservation. For observation under an optical microscope with phase contrast, the initial specimens were mounted in Heinz’s medium after clearing in Nesbitt’s fluid. To compare the effectiveness of different clearing methods, additional specimens were gently boiled in a glass evaporating dish containing 96% ethyl alcohol on an electric hot plate for approximately 1 min to remove fat from the body. For clearing, the specimens were transported to concave glass dish with 10% aqueous solution of KOH for 1 min, after which they were transferred to a dish containing chlorophenol until they became transparent. Finally, the specimens were mounted in Swann medium. Once dry, the cover glasses were sealed with nail polish or Canada balsam for protection of the mounting medium against drying. The specimens were observed in Leica DM 2500 light microscope equipped with phase and DIC contrasts, and a drawing tube.

Abbreviations for morphological term used in the text and figures are as follows:

**Abd** abdominal segment

**Ant** antennal segment

**Ant IIIO** ensory organ Ant III

**ASp** anal spines

**Cl** claws

**sm** serrate mesoseta

**ms** microsensillum

**os** subapical organite (sub-apical sensory peg)

**PAO** post-antennal organ

**S** sensillum/sensilla

**ss** sensory setae

**Tib** tibiotarsus

**VT** ventral tube

Institutional abbreviations:

**CoPJSU**Collembola collection of the Department of Zoology, Institute of Biology and Ecology, Faculty of Science, Pavol Jozef Šafárik University, Košice, Slovakia;

**ZMFUM** Zoology Museum of Ferdowsi University of Mashhad, Mashhad, Iran

## ﻿Results

### ﻿Taxonomy

**Class Collembola Lubbock, 1873**

**Order Poduromorpha Börner, 1913**

**Family Hypogastruridae Börner, 1906**

#### ﻿Genus Acherontiella

Taxon classificationAnimaliaPoduromorphaHypogastruridae

Absolon, 1913

882A93D5-978E-53AA-8973-E76B0BBCD7B3

##### Diagnosis.

Small Hypogastruridae with a fusiform habitus. White species, without eyes, post-antennal organ, retinaculum and furca. Tibiotarsi with 16–20 setae, including 1 or 2 knobbed or pointed tenent hairs. Claws with or without teeth. Setal pattern of type I, with mesosetae more or less serrated and sensory setae sometimes longer and smooth (Thibaud et al. 2004).

##### Type species.

*Acherontiella onychiuriformis* Absolon, 1913.

###### ﻿New taxa

#### ﻿ Acherontiella nezamdoosti

Taxon classificationAnimaliaPoduromorphaHypogastruridae

Mehrafrooz Mayvan & Kováč sp. nov.

57B6F3D2-0AC1-54CC-A47D-AFA77356E30B

https://zoobank.org/1206FFB3-B5C4-481B-8809-F8EA9CE3122F

**Figure 3.**
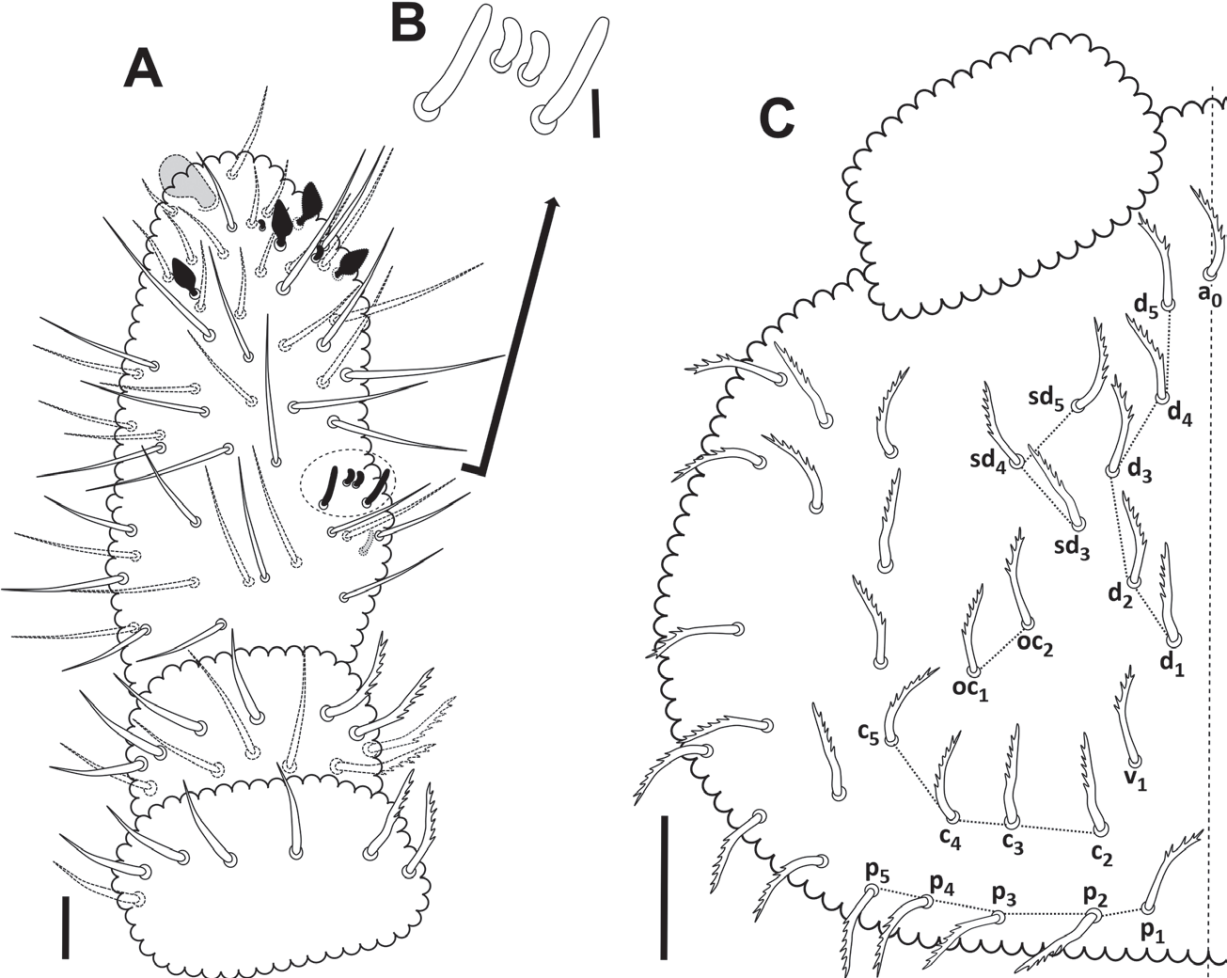
*Acherontiella nezamdoosti* sp. nov. A. Ant I–IV (right antenna), setal pattern; B. Ant III sensory organ; C. Head, setal pattern of dorsal side. Scale bars: 0.01 mm (A); 0.05 mm (B); 0.025 mm (C).

**Figure 4.**
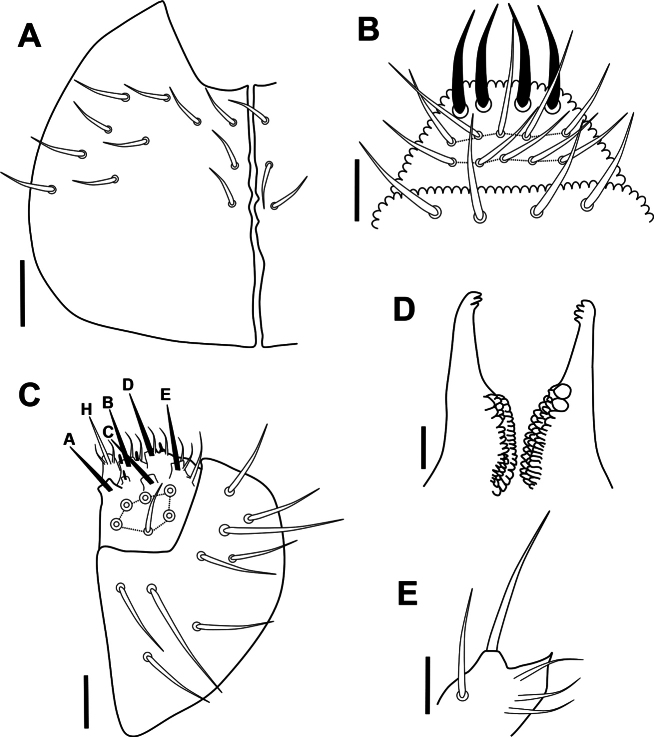
*Acherontiella nezamdoosti* sp. nov. A. Head, setal pattern of ventral side; B. Labrum, setal pattern; C. Labium and labial triangle, setal pattern; D. Mandibles; E. Maxillary outer lobe. Scale bars: 0.025 mm (A); 0.01 mm (B–E).

**Figure 5.**
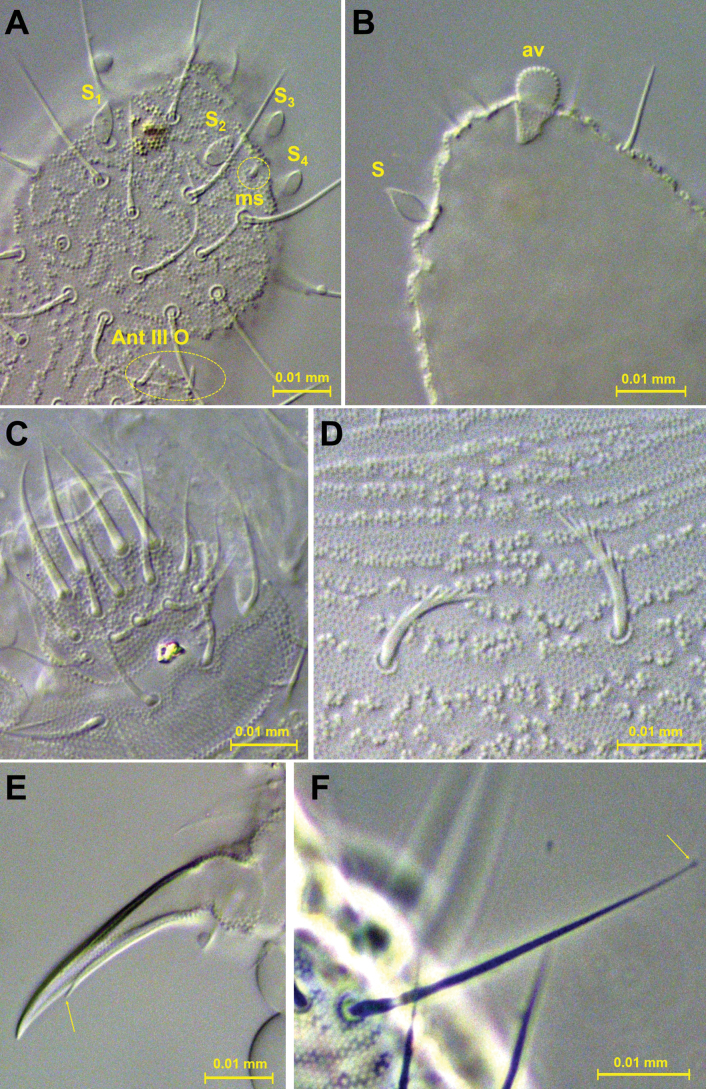
*Acherontiella nezamdoosti* sp. nov. A. Ant IV sensilla; B. Ant IV apical vesicle; C. Labral setae; D. Serrate mesosetae from Abd I; E. Claw with subapical internal tooth; F. Knobbed tenent hair on tibiotarsus. Scale bars: 0.01 mm (A–F).

**Figure 6.**
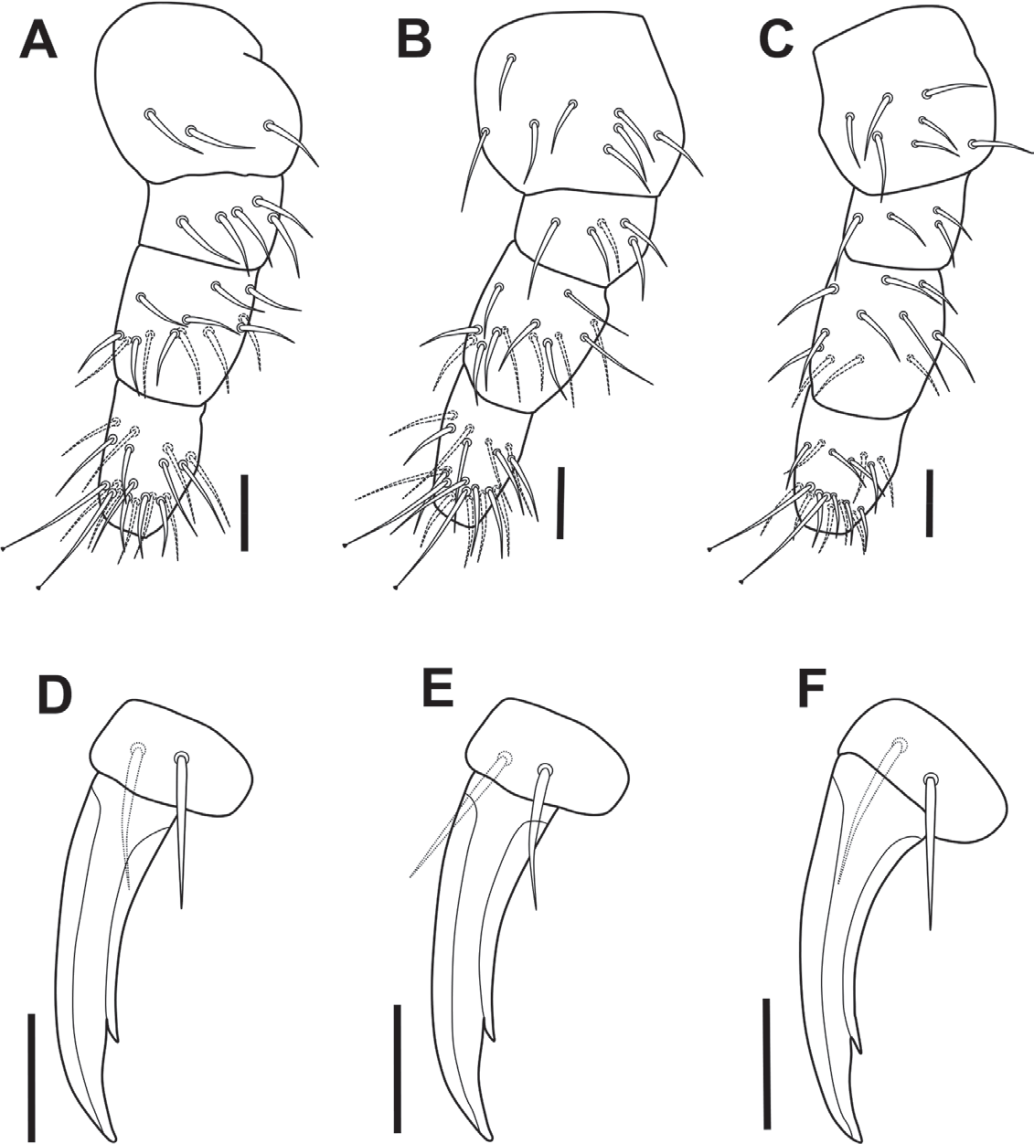
*Acherontiella nezamdoosti* sp. nov. A–C. Legs I–III, setal pattern of coxa, trochanter, femur and tibiotarsus; D–F. Legs I–III, foot complex. Scale bars: 0.02 mm (A, B); 0.01 mm (D–F).

**Figure 7.**
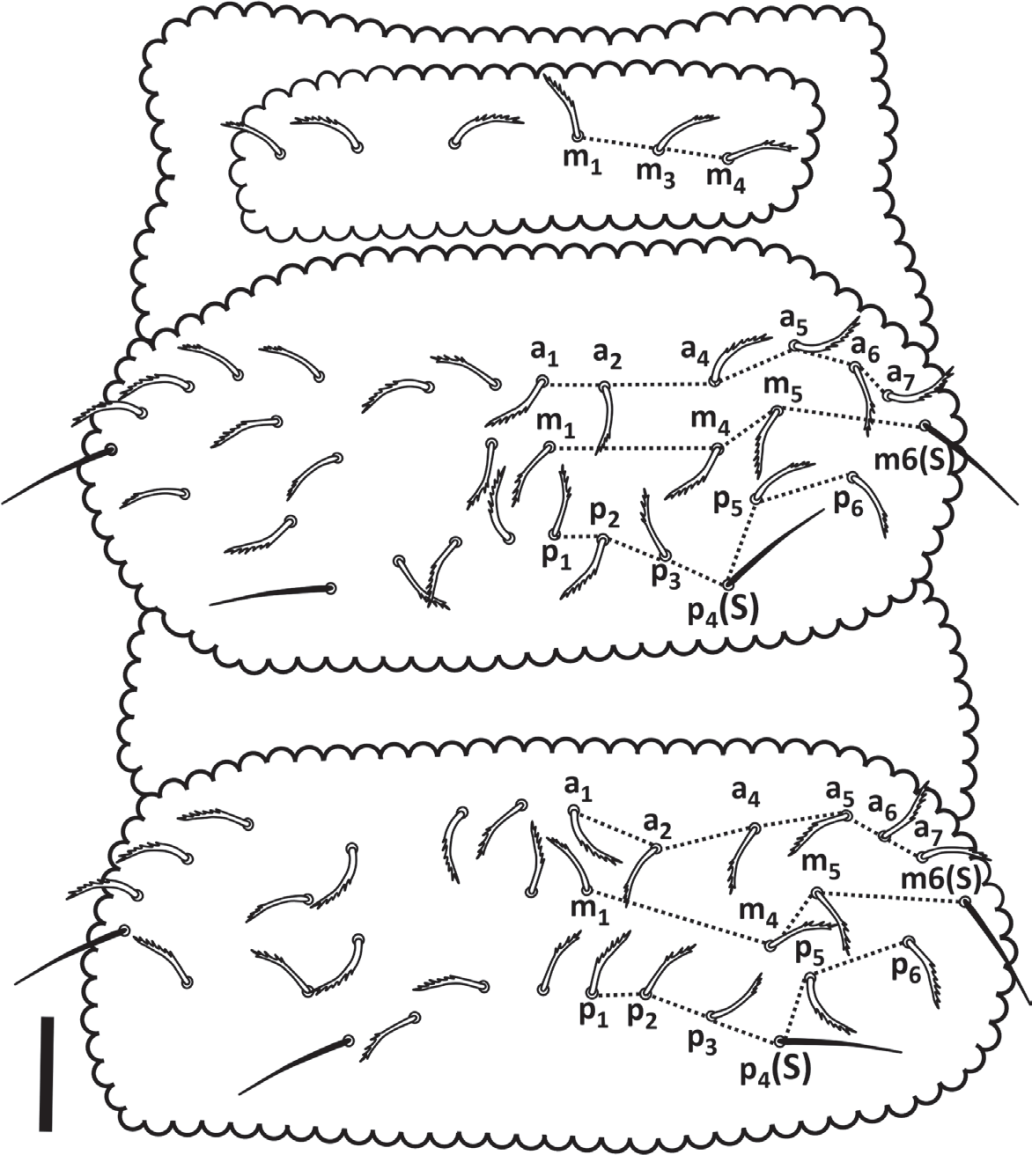
*Acherontiella nezamdoosti* sp.nov. Th I–III, setal pattern of dorsal side. Scale bar: 0.025 mm.

**Figure 8.**
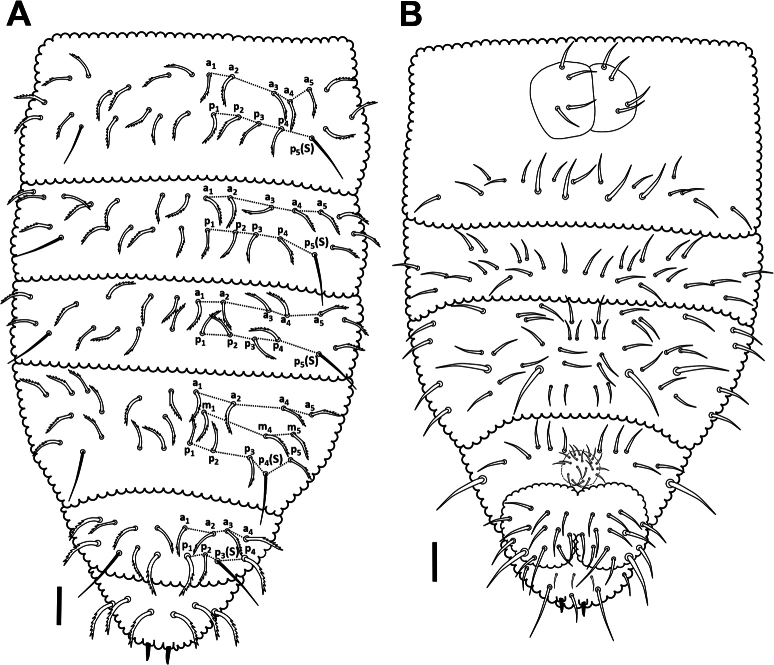
*Acherontiella nezamdoosti* sp. nov. A. Abd I–VI, setal pattern of dorsal side; B. Abd I–VI, setal pattern of ventral side. Scale bars: 0.025 mm (A, B).

Figs 3, 4, 5, 6, 7, 8, 17A, Table 2

##### Diagnosis.

Colour white, without pigment. Eyes, PAO, furca and retinaculum absent. Labrum with 4/554 setae. Four anterior setae on labrum thickened. Ant IV with simple apical vesicle and 4 subapical subglobular sensilla. VT with 4+4 apical setae. Tibiotarsi with 2 knobbed tenent hairs. Claws with strong internal subapical tooth, empodium absent. Two small anal spines present. Setae m1, m4, and m5 on Abd IV present.

##### Type material.

***Holotype*.** • ♂ on slide; Iran, Tehran province, Firouzkouh city, Herandeh village, Burnik Cave; 35°41'14"N, 52°41'24"E; 2000 m a.s.l.; 02 Aug. 2024; M. Mehrafrooz Mayvan leg.; dark zone, collected from surface of water; temperature = 8 °C, relative humidity = 87.0%; slide no. IrBuAc110. ***Paratypes.*** • 5 ♀♀, 2 juveniles, on slides; Iran, same collection data as for holotype; slide no. IrBuAc111–117; Holotype and 5 paratypes deposited in CoPJSU, 2 paratypes (IrBuAc116–117) deposited in ZMFUM.

##### Additional material.

• 4 ♂♂, 5 ♀♀, on slides; Iran, Mazandaran province, Abbas Abad County, close to the Danial village, Danial Cave; 36°39'35"N, 51°10'53"E; 203 m a.s.l.; 13 Jul. 2024; M. Mehrafrooz Mayvan leg.; dark zone, deep section of the cave, Rizan chamber, collected from surface of water; temperature = 16 °C, relative humidity = 84.0%; slide no. IrDaAc118–126; deposited in CoPJSU.

• 10 ♂♂, 8 ♀♀, on slides; Iran, North Khorasan province, Bojnourd city, close to the Bidak village, Bidak Cave; 37°26'30"N, 57°13'14"E; 1327 m a.s.l.; 23 Jun. 2022 and 26 Jul. 2023; M. Mehrafrooz Mayvan leg.; dark zone, terminal part of the cave, collected from bat guano; slide no. IrBiAc127–145; deposited in CoPJSU.

• 7 ♂♂, 3 ♀♀, on slides; Iran, Razavi Khorasan province, Sarakhs county, close to Bazangan village, Bazangan Cave; 36°18'29"N, 60°22'19"E; 1401 m a.s.l.; 22 Nov. 2021, 18 Jun. 2022 and 27 Sep. 2023; M. Mehrafrooz Mayvan leg.; dark zone, terminal part of the cave, collected from bat guano, rotten wood and pitfall traps with propylene glycol; slide no. IrBaAc146–155; deposited in CoPJSU.

• 2 ♂♂, 2 ♀♀, on slides; Iran, Razavi Khorasan province, Chenaran city, near the industrial town, Aghol Cave; 36°33'36"N, 59°04'58"E; 1445 m a.s.l.; 07 Jul. 2022 and 08 Aug. 2023; M. Mehrafrooz Mayvan leg.; dark zone, terminal part of the cave, collected from bat guano; slide no. IrAgAc156–159; deposited in CoPJSU.

• 16 ♂♂, 1 juvenile, on slides; Iran, Razavi Khorasan province, Mashhad city, Kardeh village, Kardeh Cave; 36°40'15"N, 59°40'37"E; 1458 m a.s.l.; 01 Jul. 2022, 10 Jul. 2023 and 18 Aug. 2024; M. Mehrafrooz Mayvan leg.; dark zone, collected from bat guano in Bat chamber and from rotten wood in 2^nd^ cave floor; temperature = 16 °C, relative humidity = 80.0%; slide no. IrKaAc160–176; deposited in CoPJSU.

• 5 ♂♂, 5 ♀♀, on slides; Iran, Razavi Khorasan province, Mashhad city, Kardeh village, AL Cave; 36°40'0.2"N, 59°39'19"E; 1693 m a.s.l.; 13 Oct. 2023 and 24 May 2024; M. Mehrafrooz Mayvan leg.; dark zone, collected on bat guano with an aspirator; slide no. IrAlAc177–186; deposited in CoPJSU.

• 4 ♂♂, 9 ♀♀, on slides; Iran, Razavi Khorasan province, Chenaran city, Kenan 1 (Big Kenan) Cave; 36°33'58"N, 59°04'19"E; 1451 m a.s.l.; 29 Jun. 2022 and 24 May 2024; M. Mehrafrooz Mayvan leg.; dark zone, collected on bat guano with an aspirator; slide no. IrKeAc187–199; deposited in CoPJSU.

##### Description.

***Body*.** Length 0.76 mm on average (*n* = 8, maximum length 0.84 mm). Habitus and buccal cone typical of genus *Acherontiella*. Eyes, PAO, retinaculum, and furca absent. Body fusiform, covered by serrate mesosetae; two small anal spines present (Fig. 17A). Colour white, without traces of pigment. Cuticular granulation rather uniform.

***Antennae*.** Antennae slightly shorter than head, 0.134 mm and 0.165 mm, respectively (Fig. 3A). Length of Ant I: II: III and IV (Ant III–IV fused dorsally) in holotype as 0.024: 0.026: 0.083 mm, respectively. All setae on Ant I–II serrate. Ant I with 7 setae; Ant II with 12 setae. Ant III and IV fused dorsally, intersegmental suture on ventral side well marked, 23 dorsal and 26 ventral setae, all normal, smooth (lateral setae longer than others). Ant IIIO with 2 central short and curved rods (microsensilla) flanked by 2 long guard sensilla of very characteristic constitution (Fig. 3B) (Deharveng and Díaz 1984); small ventrolateral microsensillum in a ventral pit. Ant IV with a protrusible simple ventroapical vesicle (Fig. 5B), 4 subglobular sensilla – 2 ventral (lateroexternal) and 2 dorsal (1 lateroexternal, 1 laterointernal) (Fig. 5A, B), dorsoexternal subapical ms and apical os near external sensilla (see D’Haese 2003).

***Head*.** Eyes and PAO absent. Setal formula of labrum 4/5,5,4, four anterior labral setae thickened (Figs 4B, 5C). Labium and labial triangle as in Fig. 4C, with 5 main papillae (A–E), 6 proximal smooth setae, 4 basomedial and 5 basolateral smooth setae. Maxillary outer lobe with 3 sublobal hairs (Fig. 4E). Left mandible with 3 teeth and right mandible with 4 teeth (Fig. 4D).

***Legs*.** Legs I–III with normal setae, tibiotarsi with 2 knobbed tenent hairs (Fig. 5F). Setal pattern as in Fig. 6A–C. Leg I: coxa with 3 setae, trochanter with 5 dorsal setae, femur with 13 setae, Tib with 19 setae, including 2 knobbed apical tenent hairs. Leg II: coxa with 8 setae, trochanter with 5 setae, femur with 13 setae, Tib with 19 setae, including 2 knobbed apical tenent hairs. Leg III: coxa with 7 setae, trochanter with 4 setae, femur with 11 setae, Tib with 18 setae, including 2 knobbed apical tenent hairs; Claw slender, internal edge with strong subapical tooth (Fig. 5E); empodium absent; pretarsus with 2 setae (1 internal, 1 external) shorter than half of the length of claw (Fig. 6D–F).

***Abdomen*.** Ventral tube with 4+4 apical setae. Retinaculum and furca absent. 2 small anal spines present. Ratio ASp: claw III = 0.36:1

***Setal pattern*.** Dorsal setal pattern generally of type I with mesosetae (after Jordana et al. 1997). Setae on head and body serrate (Fig. 5D), sensilla longer and smooth.

***Head*.** Dorsal setal pattern as in Fig. 3C. Seta a0 often present (absent in some specimens); d1–d5; sd3–sd5 (sd1 and sd2 absent); v1 [v2 absent]; c2–c5 (c1 absent); p1–p5; oc1–oc2 (oc3 absent). Ventral setal pattern as in Fig. 4A, with smooth setae.

***Thorax*.** Dorsal setal pattern as in Fig. 7. Position of ss in *p* row of setae on Th II–III is 4 and 4, respectively. Th I with 3+3 setae (m1, m3 and m4); Th II and III with 3 rows of setae – *a*: 4+4 as a1, a2, a4 and a5 [a3 absent]; *m*: 3+3 between m6(s) as m1, m4 and m5 [m2 and m3 absent]; *p*: 3+3 between p4(s) as p1–p3. Thoracic sterna without setae.

***Abdomen*.** Dorsal setal pattern as in Fig. 8A, ventral setal pattern as in Fig. 8B. The position of ss in *p* row on Abd I–V as 5, 5, 5, 4, 3, respectively. Abd I–III with 2 rows of setae –*a*: 5+5 as a1–a5; *p*: 4+4 between p5(s) as p1–p4. Abd IV with 3 rows of setae – *a*: 4+4 as a1, a2, a4 and a5 [a3 absent]; *m*: 3+3 as m1, m4 and m5 [m2 and m3 absent]; *p*: 3+3 between p4(s) as p1–p3. Abd V with 2 rows of setae – *a*: 3+3 as a1–a3; *p*: 2+2 between p3(s) as p1 and p2.

##### Etymology.

The new species is dedicated to Javad Nezamdoost, the founder and former director of the Cave and Speleology Association (ISCA) of Iran who has made a lot of efforts in exploration and protection of Iranian caves.

##### Taxonomic remarks.

So far, 22 species of *Acherontiella* have been described (Table 2), which may be divided into two groups based on the presence of anal spines. There are 10 *Acherontiella* species with anal spines: *A. nezamdoosti* sp. nov., *A. aokii* Tamura & Yue, 1999, *A. bougisi*, *A. cavernicola* (Tarsia in Curia, 1941), *A. epigea* Bonet, 1945, *A. mac* (Palacios-Vargas & Thibaud, 1985), *A. prominentia* Thibaud & Weiner, 1997, *A. sabina* Bonet, 1945, *A. thai* Thibaud, 1990 and *A. thibaudi* Barra, 1994. Of these, the new species along with *A. bougisi*, *A. epigea*, and *A. sabina* have claws with strong tooth, while claws in *A. aokii*, *A. cavernicola*, *A. mac*, and *A. thai* are toothless. *Acherontiella nezamdoosti* sp. nov. and *A. sabina* differ from *A. epigea* and *A. bougisi* by 2 knobbed tenent hairs on tibiotarsi, while *A. epigea* and *A. bougisi* has just 1 more or less knobbed tenent hair. Moreover, *A. nezamdoosti* sp. nov. differs from *A. sabina* in Ant IV with 4 short subglobular sensilla, Th1 with setae m1, m3, and m4 and Th II–III without setae a3, m2, and m3. In the contrary, *A. sabina* has Ant IV with 4 long cylindrical sensilla, Th1 with setae m1, m2, m4 and Th II–III with setae a3, m2, and m3. Additionally, *A. bougisi* is the only species of this genus that has been recorded from Iran so far. *Acherontiella nezamdoosti* sp. nov. clearly differs from *A. bougisi* in several diagnostic characters: 4 sensilla on Ant IV are subglobular, and fully exposed (not concealed by tegumentary folds), whereas in *A. bougisi*, they are thick, cylindrical, and partially hidden by tegumentary folds. Setae d1 and oc3 are absent on the dorsal side of head in the new species, but present in *A. bougisi*. Seta a7 (la1) is present on Th II–III in *A. nezamdoosti* sp. nov., while it is absent in *A. bougisi*. Furthermore, seta a3 is present on Abd I–III in the new species but it is absent in *A. bougisi*. The two species also differ in the *p* row of Abd IV, where *A. nezamdoosti* sp. nov. bears ss in position p4, in contrast to p5 in *A. bougisi*. Finally, seta a2 is present on Abd V in the new species, whereas it is absent in *A. bougisi*.

**Figure 9.**
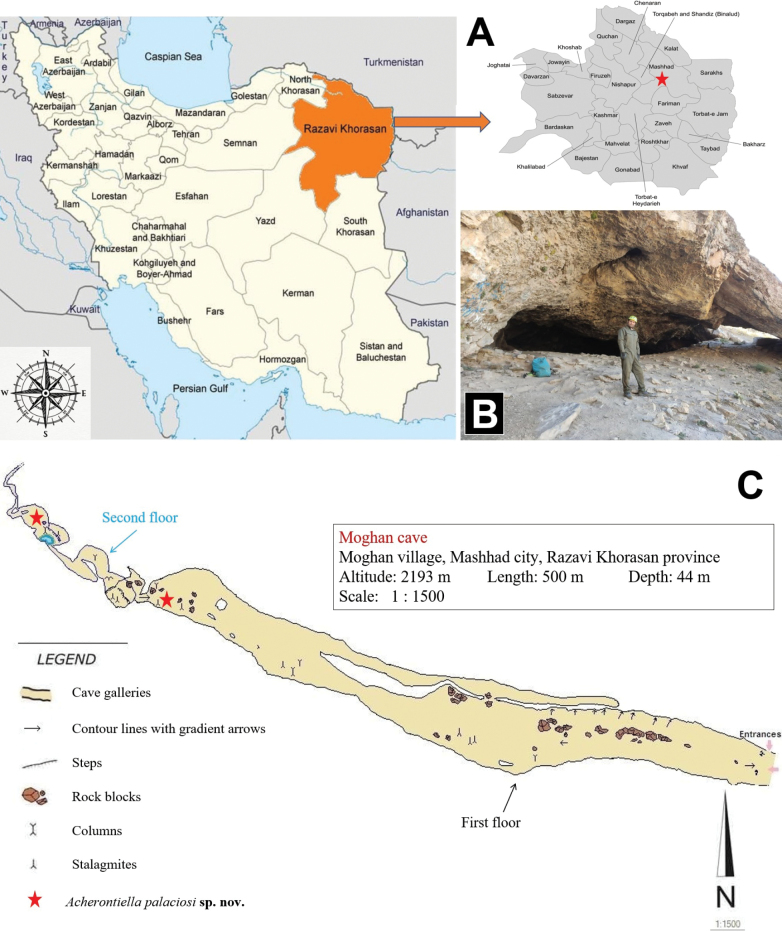
A. Location of Razavi Khorasan province in Iran (orange) and Moghan Cave (red asterisk), type locality of *A. palaciosi* sp. nov.; B. Main entrance of Moghan Cave in autumn (photo: M. Mehrafrooz Mayvan); C. Ground plan of the Moghan Cave (sketched by V. Ashrafi).

**Figure 10.**
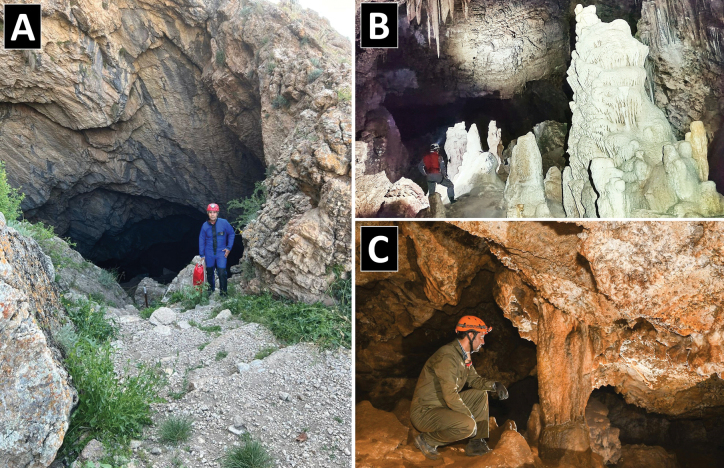
A. Burnik Cave – stairs into steep entrance and cave surroundings in spring (photo: Mahmood Mehrafrooz); B. Burnik Cave – giant flowstones in stalagmite hall (photo: H. Nezamdoost); C. Moghan Cave showing a column in the first cave floor where *A. palaciosi* sp. nov. was collected (photo: V. Ashrafi).

**Table 2.** Differential characters of world species of *Acherontiella.* Characters: 1, Anal spines: 0 = absent, 1 = present; 2, Tibiotarsal tenent hair(s) number; 3, Tibiotarsal tenent hair form: 1 = pointed, 2 = knobbed; 4, Ant IV sensilla number; 5, internal tooth on claw: 0 = absent, 1 = weekly developed, 2 = strong; 6, VT – number of apical setae; 7, Th I *m* setae; 8, Th II–III *a* row setae; 9, Th II–III *m* row setae; 10, Th II–III *p* row setae; 11, Abd I–III *a* row setae; 12, Abd I–III *p* row setae; 13, Abd IV *a* row setae; 14, Abd IV *m* row setae; 15, Abd IV *p* row setae; 16, Abd V *a* row setae; 17, Abd V *m* row setae; 18, Abd V *p* row setae. “-“= absence of data.

Species/Characters	1	2	3	4	5	6	7	8	9	10	11	12	13	14	15	16	17	18
*A. aokii* Tamura & Yue, 1999	1	0	–	4	0	4+4	1 3 4	1 3 4 5	1 3 4	1 3 4(s)	1 3 4 5	1–5(s)	1 2 5	0	1–4(s)	1 3	0	1–3(s)
*A. bougisi* Cassagnau & Delamare Deboutteville, 1955	1	1–2	2	4	1	4+4	1 3 4	1 2 4 5	1 3 5	1–4(s)	1 2 3 4 5	1–5(s)	1 2 4 5	1 3 5	1–5(s)	1 3	0	1–3 (s)
*A. candida* (Delamare Deboutteville, 1952)	0	0	–	5	0	4+4	1 3 4	1 2 4 5	1 3 5	1–4(s)	1 2 3 4 5	1–5(s)	1 2 4 5	1 3 5	1–4(s)	1 3	0	1–3(s)
*A. carusoi* Dallai, 1978	0	2	2	4	2	4+4	1 (2) 3 4	1 2 4 5	1 4 5	1 2 4(s)	1 2 4 5	1 2 3 5(s)	1 2 4 5	4	1–4(s)	1 2 3	0	1–3(s)
*A. cassagnaui* Thibaud, 1967	0	2	2	4	2	4+4	1 3 4	1 2 4 5	1 4 5	1 2 4(s)	1 2 3 4 5	1–5(s)	1 2 4 5	1 4 5	1–4(s)	1 2 3	1 3	1–3(s)
*A. cavernicola* (Tarsia in Curia, 1941)	1	2	2	4	0	4+4	–	–	–	–	–	–	–	–	–	–	0	–
*A. colotlipana* Palacios-Vargas & Thibaud, 1985	0	1	1	5	0	4+4	1 3 4	1 2 4 5	1 4 5	1–4(s)	1 2 3 4 5	1–5(s)	1 2 4 5	1 3	1–4(s)	1 3	0	1–3(s)
*A. dentata* Djanaschvili, 1971	0	2	2	4	2	4+4	1 3 4	1 2 4 5	1 4 5	1 2 4(s)	1 2 3 4 5	1–5(s)	1 2 4 5	2	1–4(s)	1 2 3	0	1–3(s)
*A. epigea* Bonet, 1945	1	1	2	4	2	4+4	1 3 4	1 2 3 4 5	1 3 4	1–4(s)	1 2 3 4 5	1–5(s)	1 2 4 5	1 3 5	1–4(s)	1 3	0	1–3(s)
*A. globulata* Thibaud & Massoud, 1980	0	1	1	4	0	4+4	1 3 4	1 2 4 5	1 3 5	1–4(s)	1 2 4 5	1–5(s)	1 2 4 5	1 4	1–4(s)	1 3	0	1–2(s)
*A. kowalskiorum* Weiner & Najt, 1998	0	0	–	5	0	4+4	1 3 4	1 2 4 5	1 4 5	1–4(s)	1 2 3 4 5	1–5(s)	1 2 4 5	1 3 5	1–5(s)	1 3	0	1–3(s)
*A. mac* (Palacios-Vargas & Thibaud, 1985)	1	1	1	4	0	4+4	1 3 4	1 2 4 5	1 4 5	1 2 4(s)	1 2 4 5	1–5(s)	1 2 4 5	3 5	1–4(s)	1 3	0	1–3(s)
*A. massoudi* Thibaud, 1963	0	2	2	8	0	4+4	1 3 4	1 2 4 5	4	1 2 4(s)	1 2 3 5	1 2 3 5(s)	1 2 4 5	3	1 2 4(s)	1 2 3	0	1–2(s)
*A. nezamdoosti* **sp. nov.**	1	2	2	4	2	4+4	1 3 4	1 2 4 5	1 4 5	1–4(s)	1 2 3 4 5	1–5(s)	1 2 4 5	1 4 5	1–4(s)	1 2 3	0	1–3(s)
*A. onychiuriformis* Absolon, 1913	0	1	1	4	0	4+4	1 3 4	1 2 4 5	1 4 5	1 2 4(s)	1 2 3 4 5	1–5(s)	1 2 4 5	1 4 5	1–4(s)	1 2 3	1 3	1–3(s)
*A. palaciosi* **sp. nov.**	0	2	2	4	2	4+4	1 4	1 2 4 5	1 4	1 2 4(s)	1 2 3 4 5	1–5(s)	1 2 4 5	4 (5)	1–4(s)	1 3	0	1–3(s)
*A. prominentia* Thibaud & Weiner, 1997	1	0	–	6	0	5+5	1 3 4	1 2 4	1 3 4	1 3 4(s)	1 2 3 5	1–5(s)	1 2 4 5	3 4	1–4(s)	1 2 3	0	1–2(s)
*A. sabina* Bonet, 1945	1	2	2	4	2	4+4	1 2 4	1 2 4 5	1 3 5	1–4(s)	1 2 3 4 5	1–6(s)	1 2 4 5	1 3 4	1–4(s)	1 2 3	0	1–3(s)
*A. thai* Thibaud, 1990	1	1	1	4	0	4+4	1 3 4	1 2 4 5	1 3 5	1–4(s)	1 3 4 5	1 2 4 5(s)	1 2 4 5	0	1 3 4(s)	1 3	0	2(s)
*A. thibaudi* Barra, 1994	1	0	–	5	0	5+5	1 3 4	1 (2) 3 4 5	1 3 4	1 2 4(s)	1 2 3 4 5	1–5(s)	1 2 4 5	3 5	1–4s)	1 3	2	1–3(s)
*A. variabilis* Delamare Deboutteville, 1948	0	2	2	4–7	0	4+4	1 3 4	1 2 4 5	II 1 (4) III (1) 4	1 2 4(s)	1 2 3 4 5	I–II 1–4(s) III 1–5(s)	1 2 4	3	1 2 4(s)	1 2 3	0	1–2(s)
*A. xenylliformis* Gisin, 1952	0	2	2	4	1	4+4	1 3 4	1 2 4 5	1 4 5	1–4(s)	1 2 3 4 5	1–5(s)	1 2 3	(3) 4	1–5(s)	1 2 3	3	1–3(s)

#### ﻿ Acherontiella palaciosi

Taxon classificationAnimaliaPoduromorphaHypogastruridae

Mehrafrooz Mayvan & Kováč sp. nov.

77ACE6A0-D884-5177-8865-5CCD1C98B4AF

https://zoobank.org/9E29ADBC-3F97-48B9-9F54-49C8DA27B59E

**Figure 11.**
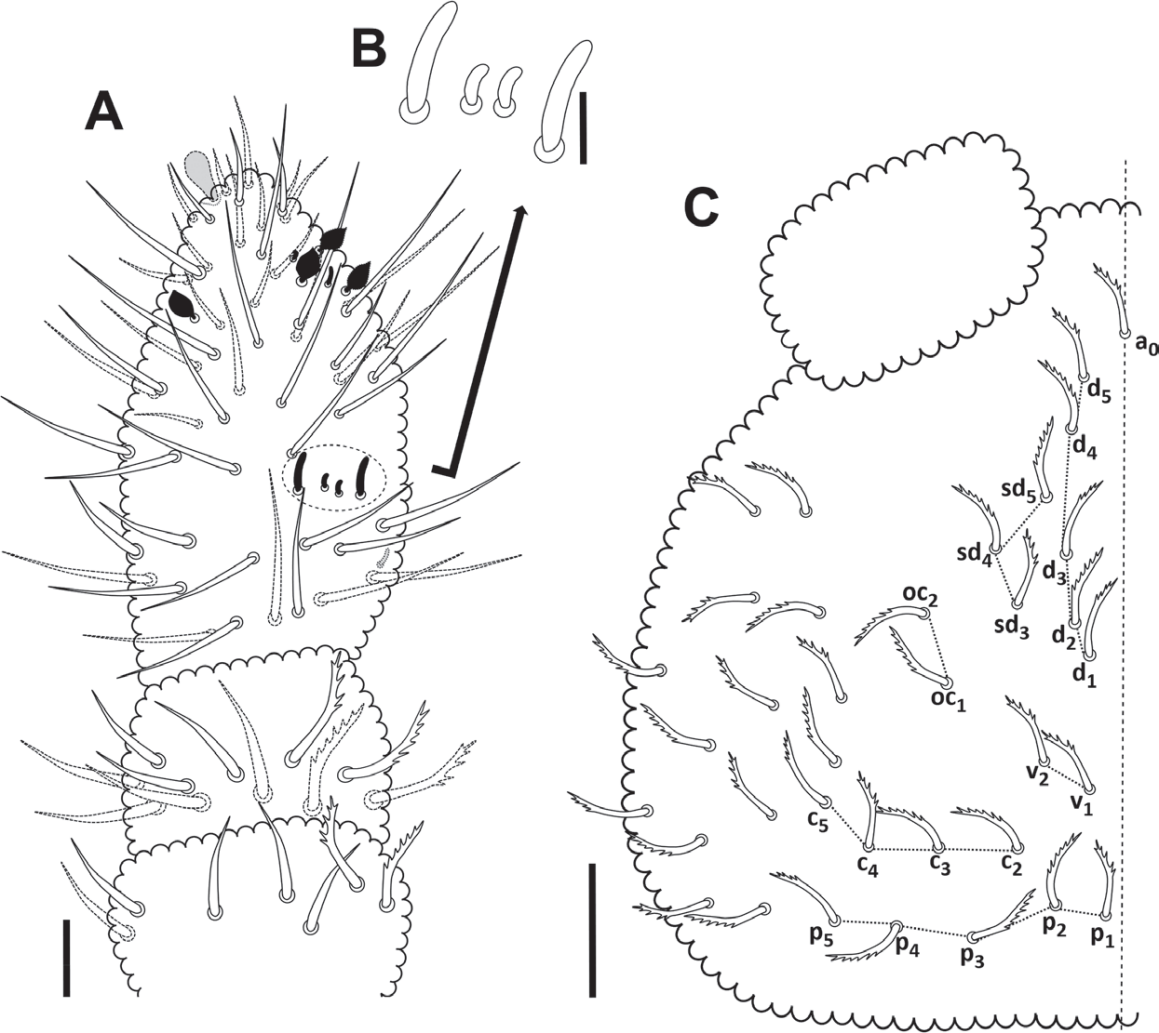
*Acherontiella palaciosi* sp. nov. A. Ant I–IV (left antenna), setal pattern on ventral side; B. Ant III sensory organ; C. Head, setal pattern of dorsal side. Scale bars: A = 0.02 mm (N); B = 0.05 mm (N); C = 0.025 mm (N).

**Figure 12.**
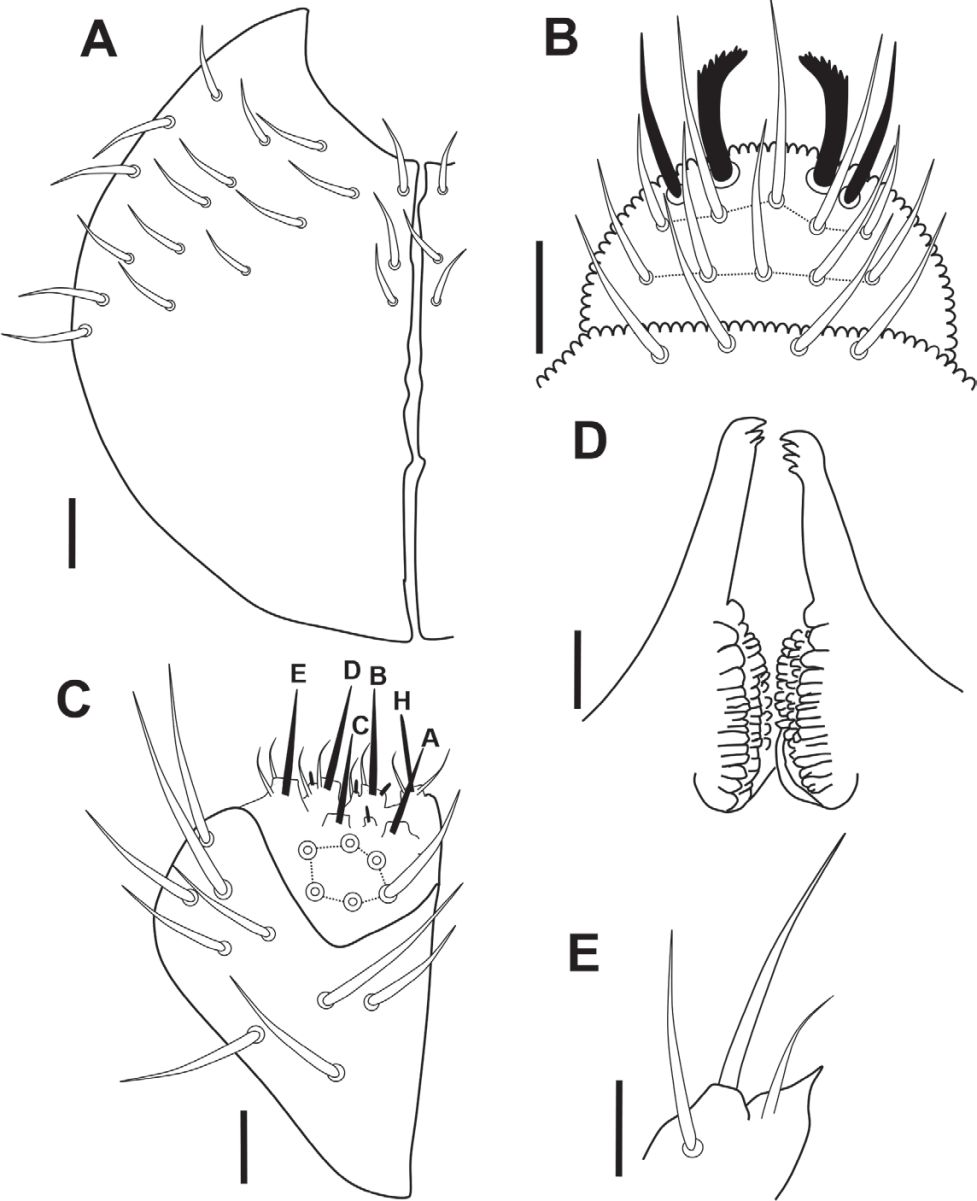
*Acherontiella palaciosi* sp. nov. A. Head, setal pattern on ventral side; B. Labrum, setal pattern; C. Labium and labial triangle, setal pattern; D. Mandibles; E. Maxillary outer lobe. Scale bars: 0.025 mm (A); 0.01 mm (B–E).

**Figure 13.**
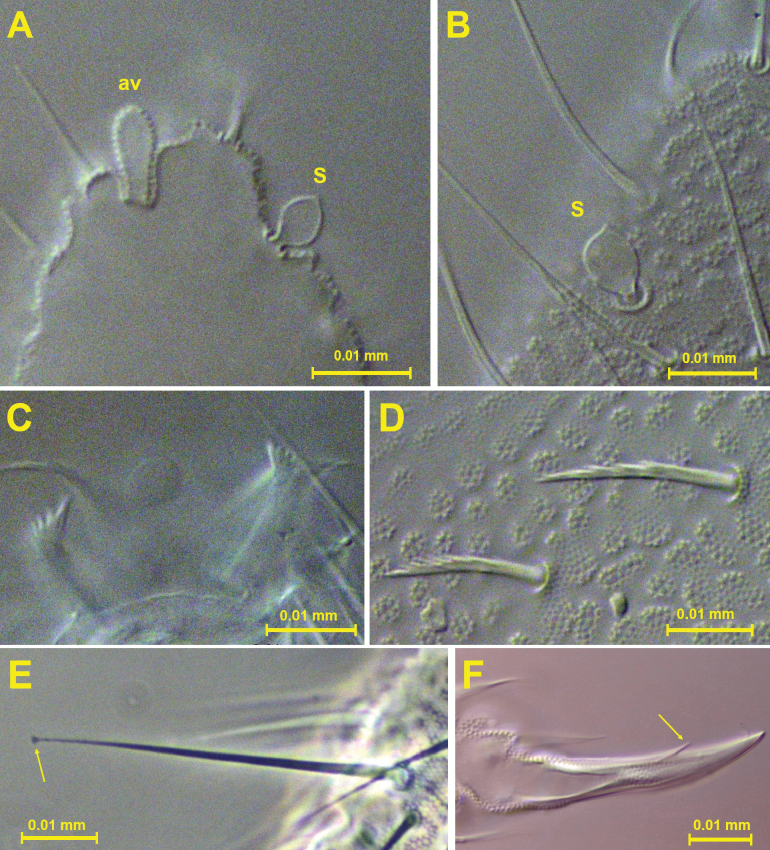
*Acherontiella palaciosi* sp. nov. A. Ant IV apical vesicle; B. Ant IV sensillum; C. Two medial setae in anterior row of labrum; D. Serrate mesosetae of Th II; E. Knobbed tenent hair on tibiotarsi; F. Claw with subapical tooth. Scale bars: 0.01 mm (A–F).

**Figure 14.**
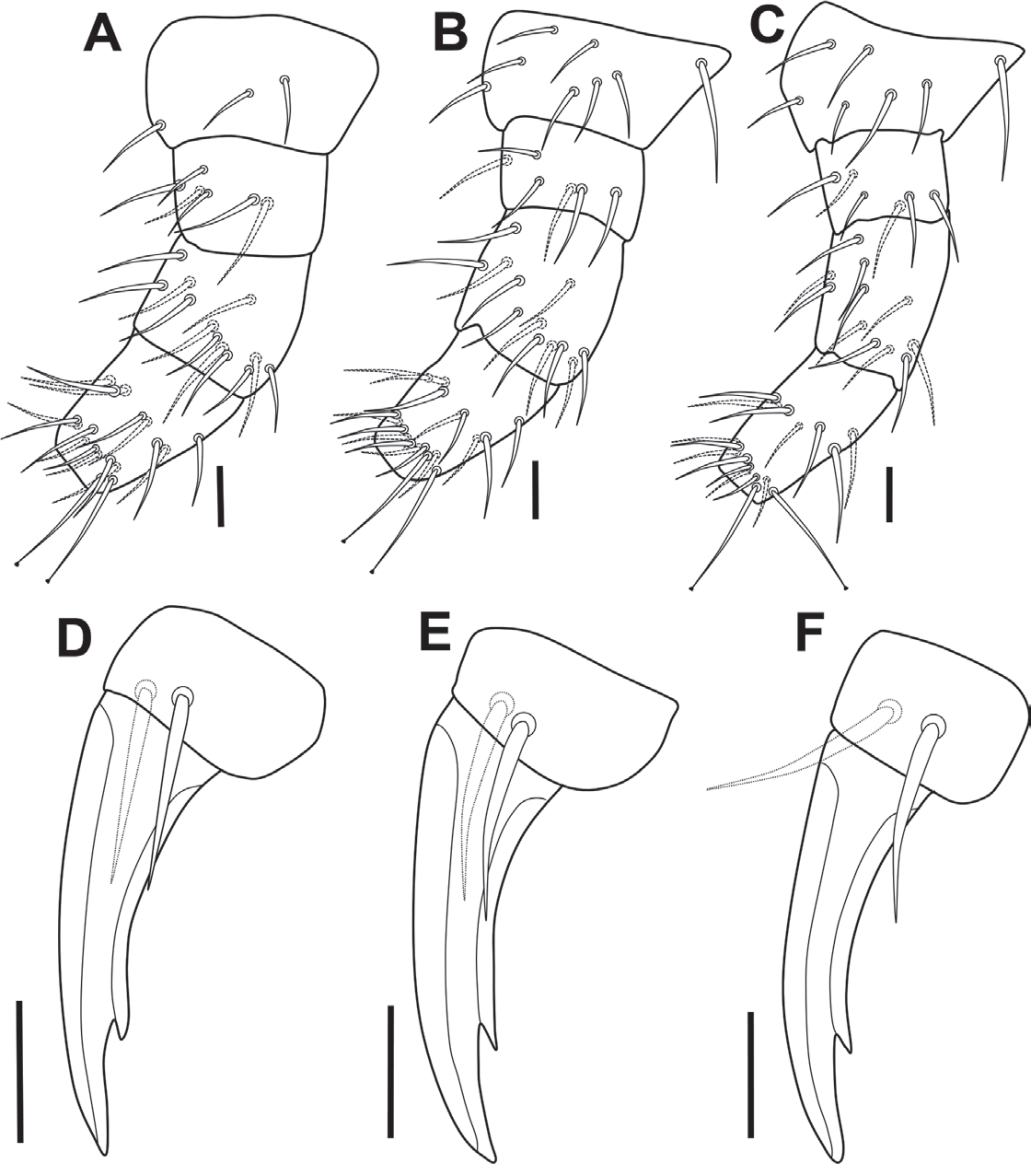
*Acherontiella palaciosi* sp. nov. A–C. Legs I–III, setal pattern of coxa, trochanter, femur and tibiotarsus; D–F. Legs I–III, foot complex. Scale bars: 0.01 mm (A–F).

**Figure 15.**
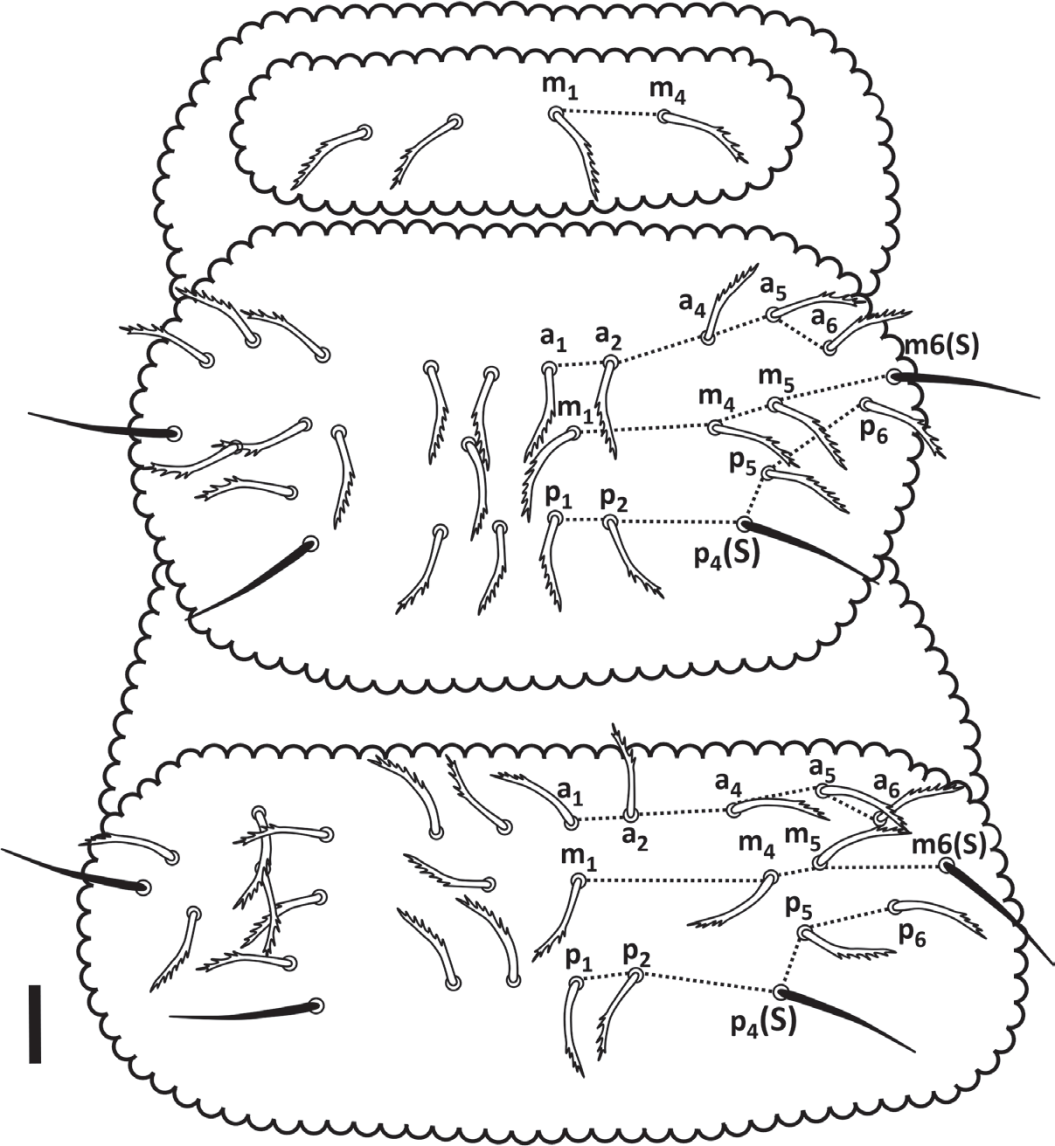
*Acherontiella palaciosi* sp.nov. Th I–III, setal pattern of dorsal side. Scale bar: 0.025 mm.

**Figure 16.**
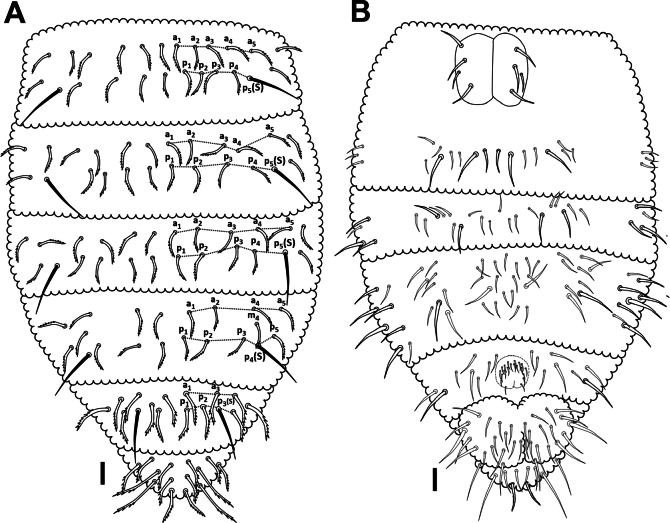
*Acherontiella palaciosi* sp.nov. A. Abd I–VI setal pattern of dorsal side; B. Abd I–VI setal pattern of ventral side. Scale bars: 0.025 mm (A, B).

**Figure 17.**
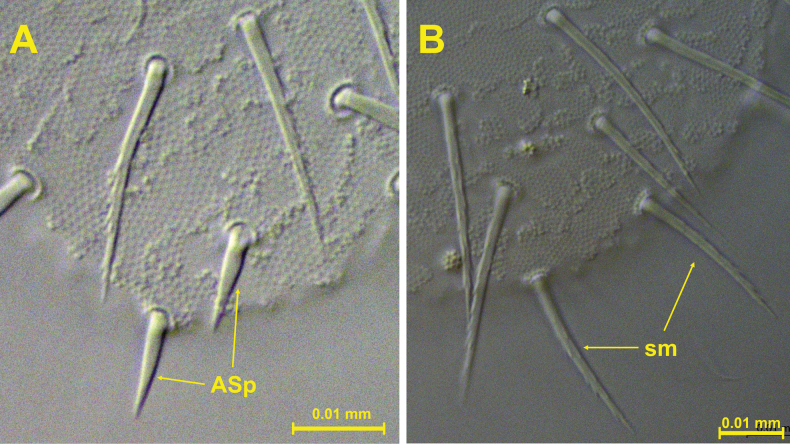
A. *Acherontiella nezamdoosti* sp. nov. – two small anal spines on Abd VI; B. *Acherontiella palaciosi* sp. nov. – two serrate mesosetae in position of anal spines on Abd VI. Scale bars: 0.01 mm (A, B).

Figs 11, 12, 13, 14, 15, 16, 17B, Table 2

##### Diagnosis.

Eyes, PAO, furca, and retinaculum absent. Labrum with 4/554 setae, anterior row with 2 medial apically dentate spine-like setae. Ant IV with simple apical vesicle and 4 subapical globular sensilla. VT with 4+4 apical setae. Tibiotarsi with 2 knobbed tenent hairs. Claws with strong subapical internal tooth, empodium absent. Anal spines absent. Th I with setae m1 and m4.

##### Type material.

**Holotype.** • ♂, on slide; Iran, Razavi Khorasan province, Mashhad city, Moghan village, Moghan Cave; 36°06'59"N, 59°22'06"E; 2193 m a.s.l.; 01 Aug. 2023; M. Mehrafrooz Mayvan leg.; dark zone, deep section of the cave, collected from rotten wood in 2^nd^ cave floor and from a stalagmite column in 1^st^ floor; temperature = 9 °C, relative humidity = 84.0%; slide no. IrMoAc101. **Paratypes.** • 4 ♂♂, 4 ♀♀, 1 juvenile, on slides; Iran, same collection data as for holotype; slide no. IrMoAc 102–109; Holotype and 8 deposited in CoPJSU, 1 paratype (IrMoAc 109) deposited in ZMFUM.

##### Description.

***Body*.** Length 1.0 mm on average (*n* = 9, maximum length 1.18 mm). Habitus and buccal cone typical of the genus *Acherontiella*. Eyes, PAO, retinaculum, and furca absent. Body fusiform, covered by serrate mesosetae (Fig. 13D), anal spines absent. Colour white, without traces of pigment. Cuticular granulation rather uniform.

***Antenna*e.** Antennae slightly shorter than head, 0.18 mm and 0.20 mm, respectively (Fig. 11A). Length of Ant I: II: III and IV (dorsally fused) as 0.034: 0.036: 0.11 mm, respectively. All setae on Ant I–II slightly serrate. Ant I with 7 setae; Ant II with 11 setae. Ant III and IV dorsally fused, intersegmental suture on ventral side well marked, 26 dorsal and 25 ventral setae, all normal, smooth (lateral setae longer than other setae). Ant IIIO with 2 central short and curved rods (microsensilla) flanked by 2 long guard sensilla of characteristic constitution (Deharveng and Díaz 1984) (Fig. 11B), a small ventrolateral microsensillum in a ventral pit. Ant IV with a protrusible ventroapical simple vesicle (Fig. 13A), 4 globular sensilla (Fig. 13B) – 2 dorsal (1 lateroexternal, 1 laterointernal) and 2 ventral (both lateroexternal), dorsoexternal subapical ms and apical os near external sensilla (see D’Haese 2003).

***Head*.** Eyes and PAO absent. Setal formula of labrum 4/5,5,4, two medial setae in anterior row on labrum thickened as spines and apically dentate (Figs 12B, 13C). Labium and labial triangle as in Fig. 12C, with 5 main papillae (A–E), 6 proximal smooth setae, 4 basomedial and 5 basolateral smooth setae. Maxillary outer lobe with 1 sublobal hair (Fig. 12E). Left mandible with 3 teeth and right mandible with 4 teeth (Fig. 12D).

***Legs*.** Legs I–III with normal setae, tibiotarsi with 2 knobbed tenent hairs (Fig. 13E). Setal pattern as in Fig. 14A–C. Leg I: coxa with 3 setae, trochanter with 6 setae, femur with 12 setae, Tib with 18, including 2 knobbed apical tenent hairs. Leg II: coxa with 8 setae, trochanter with 6 setae, femur with 12 setae, Tib with 18, including 2 knobbed apical tenent hairs. Leg III: coxa with 7 setae, trochanter with 6 setae, femur with 11 setae, Tib with 17, including 2 knobbed apical tenent hairs; claw slender (Fig. 14D–F), internal edge with strong subapical tooth (Fig. 13F); empodium absent. Pretarsus with 2 setae (1 internal, 1 external), both reaching the half length of internal lamella.

***Abdomen*.** Ventral tube with 4+4 setae. Retinaculum and furca absent. Anal spines absent, substituted by 2 serrate setae (Fig. 17B).

***Setal pattern*.** Dorsal setal pattern generally of type I with mesosetae (after Jordana et al. 1997). Setae on head and body slightly serrate, sensory setae longer and smooth.

***Head*.** Dorsal setal pattern as in Fig. 11C. Seta a0 present; d1–d5; sd3–sd5 (sd1 and sd2 absent); v1–v2; c2–c5 (c1 absent); p1–p5; oc1–oc2 (oc3 absent). Ventral setal pattern as in Fig. 12A, all setae smooth.

***Thorax*.** Dorsal setal pattern as in Fig. 15. Position of ss in *p* row on Th II–III is 4 and 4, respectively. Th I with setae m1 and m4 [m2 and m3 absent]; Th II and III with 3 rows of setae – *a*: 4+4 as a1, a2, a4 and a5 [a3 absent]; *m*: 3+3 between m6(s) as m1, m4 and m5 [m2 and m3 absent]; *p*: 2+2 between p4(s) as p1 and p2 [p3 absent]. Thoracic sterna without setae.

***Abdomen*.** Dorsal setal pattern as in Fig. 16A. Position of ss in *p* row on Abd I–V is 5, 5, 5, 4 and 3, respectively. Abd I–III with 2 rows of setae – *a*: 5+5 as a1–a5; *p*: 4+4 between p5(s) as p1–p4. Abd IV with 3 rows of setae – *a*: 3+3 as a1, a2 and a4 [a3 absent]; *m*: 1+1 (or 2+2) as m4 (m5) [m1, m2 and m3 absent]; *p*: 3+3 between p4(s) as p1–p3. Abd V with 2 rows of setae – *a*: 3+3 as a1, a3 and a4 [a2 absent]; *p*: 2+2 between p3(s) as p1–p2. Ventral abdominal setal pattern as in Fig. 16B.

##### Etymology.

The new species is dedicated to our colleague and friend professor José G. Palacios-Vargas (Universidad Nacional Autónoma de México) for his immense contribution to taxonomy of soil and subterranean Collembola.

##### Taxonomic remarks.

*Acherontiella palaciosi* sp. nov. is similar to 11 species in lack of anal spines: *A. candida* (Delamare Deboutteville, 1952), *A. carusoi* Dallai, 1978, *A. cassagnaui* Thibaud, 1967, *A. colotlipana* Palacios-Vargas & Thibaud, 1985, *A. dentata* Djanaschvili, 1971, *A. globulata* Thibaud & Massoud, 1979, *A. kowalskiorum* Weiner & Najt, 1998, *A. massoudi* Thibaud, 1963, *A. onychiuriformis* Absolon, 1913, *A. variabilis* Delamare Deboutteville, 1948, and *A. xenylliformis* Gisin, 1952. Among them, *A. palaciosi* sp. nov., *A. carusoi*, *A. cassagnaui*, *A. dentata*, *A. massoudi*, *A. variabilis*, and *A. xenylliformis* differ from others by 2 knobbed tenent hairs on tibiotarsi, while the rest of the species have 1–2 sharp tenent hairs on tibiotarsi. Moreover, claws in *A. palaciosi* sp. nov., *A. carusoi*, *A. cassagnaui* and *A. dentata* are equipped with a strong subapical tooth; however, claws are toothless in *A. massoudi*, *A. variabilis*, and *A. xenylliformis*. In *A. palaciosi* sp. nov., *A. cassagnaui*, and *A. dentata*, 2 medial setae in anterior row on labrum are thickened as spines and apically dentate, while in *A. carusoi* all 4 anterior labral setae are sharp and smooth. *Acherontiella palaciosi* sp. nov. differs from *A. cassagnaui* by Th I with 2+2 *m* setae, absence of seta a3 on Abd IV, presence of seta m4 (m5) on Abd IV, and absence of seta a2 and *m* setae on Abd V. In *A. cassagnaui* 3+3 *m* setae on Th I and seta a3 on Abd IV are present, as well as setae m1 (m3) and m4 on Abd IV and seta a2 and *m* setae on Abd V. Finally, *A. palaciosi* sp. nov. differs from *A. dentata* by following characters: setae a0 and sd3 are present, while oc3 is absent on dorsal side of head, maxillary outer lobe with one sublobal hair, seta m3 on Th I is absent, seta m4 on Abd IV is present, and seta a2 on Abd V is absent. In contrast, in *A. dentata* setae a0 and sd3 are absent, while oc3 is present on the dorsal side of head; maxillary outer lobe with 2 sublobal hairs, seta m3 on Th I is present, Abd IV with seta m2 in the *m* row, and seta a2 on Abd V is present.

### ﻿Identification key to world species of the genus *Acherontiella*

The following identification key is based on the diagnostic characters of all described *Acherontiella* species (adapted from Dallai 1978; Bonet 1945; Thibaud 1990; Thibaud et al. 2004).

**Table:** 

1	ASp absent	**2**
–	ASp present	**13**
2	Tib without or with 1 pointed tenent hair	**3**
–	Tib with 2 more or less knobbed tenent hairs	**7**
3	Ant IV with 4 sensilla	**4**
–	Ant IV with 5 sensilla	**5**
4	Two anterior medial labral setae thickened to dentate spines. Abd V with 1–3 setae in *m* row. Cl generally without teeth, sometimes with a small ventroapical internal tooth	***A. onychiuriformis* Absolon, 1913 (Algeria, cave)**
–	Anterior labral setae normal. Abd V without setae in *m* row. Cl without teeth	***A. globulata* Thibaud & Massoud, 1979 (Marie-Galante Island, cave)**
5	Tib with 1 pointed tenent hair. Tib I–III with 18, 18, and 17 setae, respectively. Subcoxae “2” with 1, 2, and 2 setae	***A. colotlipana* Palacios-Vargas & Thibaud, 1985 (Mexico, cave)**
–	Tib without tenent hair. Tib I–III with 19, 19, and 18 setae, respectively. Subcoxae “2” with 1, 2, and 3 setae	**6**
6	Abdominal sternum IV without seta m1	***A. candida* (Delamare Deboutteville, 1952) (France, soil)**
–	Abdominal sternum IV with seta m1	***A. kowalskiorum* Weiner & Najt, 1998 (Kenya, soil)**
7	Cl with strong subapical tooth	**8**
–	Cl without teeth or with 1 weakly developed tooth	**11**
8	Two labral anterior medial setae thickened into dentate spines	**9**
–	Four labral anterior setae smooth and equally long and thick	***A. carusoi* Dallai, 1978 (Italy, cave)**
9	Abd IV without seta m1. Abd V without setae in *m* row	**10**
–	Abd IV with seta m1. Abd V with 1–3 setae in *m* row	***A. cassagnaui* Thibaud, 1967 (France & Romania, cave)**
10	Dorsal head without setae a0 and sd3. Th I with seta m3. Maxillary outer lobe with 2 sublobal hairs. Abd V with seta a2	***A. dentata* Djanaschvili, 1971 (Azerbaijan, cave)**
–	Dorsal head with setae a0 and sd3. Th I without seta m3. Maxillary outer lobe with 1 sublobal hair. Abd V without seta a2	***A. palaciosi* sp. nov. (Iran, cave)**
11	Four labral anterior setae smooth and equally long and thick	***A. massoudi* Thibaud, 1963 (Ivory coast, soil)**
–	Two labral anterior medial setae thickened into dentate spines	**12**
12	Ant IV with 4–7 sensilla. Th II–III with seta m1 in *m* row. Cl without teeth	***A. variabilis* Delamare Deboutteville, 1948 (France, soil)**
–	Ant IV with 4 sensilla. Th II–III without seta m1 in *m* row. Cl with 1 small internal tooth developed at 2/3 of the lamella	***A. xenylliformis* Gisin, 1952 (Morroco, Portugal & Spain, cave)**
13	Cl with a strong tooth	**14**
–	Cl without teeth	**17**
14	Tib with 2 knobbed tenent hairs	**15**
–	Tib with 1 more or less knobbed tenent hair	**16**
15	Ant IV with 4 short subglobular sensilla. Th1 with setae m1, m3, and m4 in *m* row. Abd I–III with ss in position p5	***A. nezamdoosti* sp. nov. (Iran, cave)**
–	Ant IV with 4 elongated cylindrical sensilla. Th1 with setae m1, m2, and m4 in *m* row. Abd I–III with ss in position p6	***A. sabina* Bonet, 1945 (Mexico & USA, cave)**
16	Ant IV with 4 cylindrical and thick sensilla, partially hidden by tegumentary folds. Abd IV with setae m1, m3, and m4 in *m* row	***A. bougisi* Cassagnau & Delamare Deboutteville, 1955 (Lebanon, soil & cave)**
–	Ant IV with 4 cylindrical curved sensilla not concealed by tegumentary folds. Abd IV with setae m1, m4, and m5 in *m* row	***A. epigea* Bonet, 1945 (Mexico, cave)**
17	Tib with 2 more or less knobbed tenent hairs	***A. cavernicola* (Tarsia in Curia, 1941) (Italy, cave)**
–	Tib without or with 1 pointed tenent hair	**18**
18	Ant IV with 6 sensilla. VT with 5+5 setae	**19**
–	Ant IV with 4 sensilla. VT with 4+4 setae	**20**
19	Apical vesicle on Ant IV trilobed. Abd V with ss in position p2	***A. prominentia* Thibaud & Weiner, 1997 (New Caledonia Island, soil)**
–	Apical vesicle on Ant IV simple. Abd V with ss in position p3	***A. thibaudi* Barra, 1994 (South Africa, soil)**
20	Four labral anterior setae thickened into dentated spines. Abd V with ss in position p2	***A. thai* Thibaud, 1990 (Thailand, cave)**
–	Four labral anterior setae not dentate. Abd V with ss in position p3	**21**
21	Tib I–III with 19, 19, and 18 setae, respectively. Ant I with 7 setae. Four labral anterior setae sharp, smooth and equal	***A. mac* (Palacios-Vargas & Thibaud, 1985) (Mexico, soil)**
–	Tib I–III with 15, 15, and 14 setae, respectively. Ant I with 6 setae. Four labral anterior setae stout	***A. aokii* Tamura & Yue, 1999 (China, soil)**

## ﻿Discussion

*Acherontiella* is a cosmopolitan genus of the family Hypogastruridae (Poduromorpha) including both edaphic and subterranean species. Of the 22 known species of *Acherontiella* worldwide, 13 are cave-dwelling and eight are soil-dwelling. Notably, *A. bougisi* is the only species that has been collected from environments both within and outside of caves (Cassagnau and Delamare Deboutteville 1955; Bellinger et al. 1996–2025). Among the subterranean species, eight species, including *A. nezamdoosti* sp. nov., *A. cassagnaui*, *A. colotlipana*, *A. globulata*, *A. onychiuriformis*, *A. thai*, *A. variabilis*, and *A. xenylliformis* have been found on bat guano. This is in contrast to *A. nezamdoosti* sp. nov. and *A. palaciosi* sp. nov., which have been found on rotten wood in Moghan Cave with a general lack of guano. Unfortunately, due to the excessive number of visitors to this cave in different seasons and the lack of proper protection laws, the population of bats in this cave has become very rare. Also, *A. nezamdoosti* sp. nov. is limited to deep sections of caves and, thus, can be considered to be a troglobiotic species.

## Supplementary Material

XML Treatment for Acherontiella

XML Treatment for Acherontiella nezamdoosti

XML Treatment for Acherontiella palaciosi

## ﻿Additional information

### Use of AI

No use of AI was reported.

### Author ORCIDs

Mahmood Mehrafrooz Mayvan https://orcid.org/0000-0002-7494-0981

Ľubomír Kováč https://orcid.org/0000-0001-8194-2128

### Data availability

All of the data that support the findings of this study are available in the main text.
